# Protective Effect of Melatonin against Oxidative Stress-Induced Apoptosis and Enhanced Autophagy in Human Retinal Pigment Epithelium Cells

**DOI:** 10.1155/2018/9015765

**Published:** 2018-08-05

**Authors:** Chih-Chao Chang, Tien-Yi Huang, Hsin-Yuan Chen, Tsui-Chin Huang, Li-Chun Lin, Yen-Jui Chang, Shih-Min Hsia

**Affiliations:** ^1^School of Nutrition and Health Sciences, Taipei Medical University, Taipei, Taiwan; ^2^PhD Program for Cancer Biology and Drug Discovery, College of Medical Science and Technology, Taipei Medical University and Academia Sinica, Taipei, Taiwan; ^3^Department of Ophthalmology, Yangming Branch, Taipei City Hospital, Taipei, Taiwan; ^4^Department of Optometry, University of Kang Ning, Taipei, Taiwan; ^5^Department of Health and Welfare, College of City Management, University of Taipei, Taipei, Taiwan; ^6^Graduate Institute of Metabolism and Obesity Sciences, Taipei Medical University, Taipei, Taiwan; ^7^School of Food and Safety, Taipei Medical University, Taipei, Taiwan; ^8^Nutrition Research Center, Taipei Medical University Hospital, Taipei, Taiwan

## Abstract

Age-related macular degeneration (AMD) affects the retinal macula and results in loss of vision, and AMD is the primary cause of blindness and severe visual impairment among elderly people worldwide. AMD is characterized by the accumulation of drusen in the Bruch's membrane and dysfunction of retinal pigment epithelial (RPE) cells and photoreceptors. The pathogenesis of AMD remains unclear, and no effective treatment exists. Accumulating evidence indicates that oxidative stress plays a critical role in RPE cell degeneration and AMD. Melatonin is an antioxidant that scavenges free radicals, and it has anti-inflammatory, antitumor, and antiangiogenic effects. This study investigated the antioxidative, antiapoptotic, and autophagic effects of melatonin on oxidative damage to RPE cells. We used hydrogen peroxide (H_2_O_2_) to stimulate reactive oxygen species production to cause cell apoptosis in ARPE-19 cell lines. Our findings revealed that treatment with melatonin significantly inhibited H_2_O_2_-induced RPE cell damage, decreased the apoptotic rate, increased the mitochondrial membrane potential, and increased the autophagy effect. Furthermore, melatonin reduced the Bax/Bcl-2 ratio and the expression levels of the apoptosis-associated proteins cytochrome c and caspase 7. Additionally, melatonin upregulated the expression of the autophagy-related proteins LC3-II and Beclin-1 and downregulated the expression of p62. Thus, melatonin's effects on autophagy and apoptosis can protect against H_2_O_2_-induced oxidative damage in human RPE cells. Melatonin may have multiple protective effects on human RPE cells against H_2_O_2_-induced oxidative damage.

## 1. Introduction

Age-related macular degeneration (AMD) is the leading cause of vision loss among elderly people in developed countries [1]. Approximately 50 million people experience AMD symptoms, and 14 million people are visually impaired because of AMD [2]; the prevalence of AMD is also increasing [3]. Abnormalities in retinal pigment epithelial (RPE) cells cause vision loss in patients with AMD, and impairment of normal physiological function in RPE cells is an early pathogenesis of AMD [4].

A growing body of evidence indicates that oxidative stress plays a critical role in RPE degeneration in AMD [5]. RPE cells are particularly vulnerable to oxidative stress caused by reactive oxygen species (ROS) [6] such as superoxide anion radicals, hydroxyl radicals, singlet oxygen, and hydrogen peroxide (H_2_O_2_) [7, 8]. Research has revealed that several antioxidants and zinc-containing supplements can inhibit AMD progression and preserve vision [7, 9]. Therefore, protecting RPE cells by limiting oxidative stress may represent an effective approach to slowing or possibly reversing vision loss in patients with AMD.

In cellular systems, ROS are detrimental to cell survival; however, they are essential for cell signaling and regulation of cell proliferation, migration, differentiation, and gene expression [10, 11]. Inhibiting ROS-induced RPE cell damage may inhibit AMD progression [12, 13]. Catalase, superoxide dismutase (SOD), and glutathione peroxidase (GPx) are major enzymes that protect RPE cells through increased expression by effectively scavenging ROS and attenuating oxidative damage [14, 15]. Malondialdehyde (MDA) is a product of lipid peroxidation, and its expression is generally used as a marker of lipid peroxidation and oxidative damage [16].

Apoptosis is a form of programmed cell death, and pathological apoptosis is associated with AMD [17, 18]. A previous study reported that the antiapoptotic Bcl-2 family proteins and apoptotic Bax proteins play important roles in mitochondrion-dependent extrinsic and intrinsic cell death pathways [19, 20]. Bax could release cytochrome c from the mitochondria into the cytosol to active caspase result in apoptosis [19, 20]. Moreover, caspase 7, Bax, Bcl-2, and cytochrome c are apoptotic markers in apoptosis.

Autophagy removes damaged organelles and protein aggregates of RPE cells, which is a crucial function because these cells are exposed to oxidative stress [21]. Studies have reported that autophagy occurs in RPE cells [22, 23]. However, failure or impairment of autophagy in RPE cells may cause an accumulation of aggregation-prone proteins, cellular degeneration, and finally the induction of cell death, all of which have been related to the pathogenesis of AMD [21, 23, 24]. In addition, research revealed that the preservation of autophagic activity is vital for preventing detrimental intracellular accumulation of damaged molecules [23]. Nevertheless, whether the autophagic pathway has a protective effect on RPE cells remains unclear; moreover, the relationship between oxidative stress and autophagy and how their interaction influences RPE cells require further clarification.

Melatonin (N-acetyl-5-methoxytryptamine) is a tryptophan-derived neurohormone that performs critical functions in the regulation of many physiological systems and processes including the circadian rhythm, immune system, cardiovascular system, and aging process [25, 26]. A previous study mentions that the person to receive 250 mg melatonin every 6 h for 25–30 days shows no toxic effects [27]. A study reported that the level of urinary melatonin was relatively low in patients with AMD [28]. Therefore, studying the relationship between the effects of melatonin and AMD may be constructive. Melatonin is mainly synthesized and secreted by the pineal gland [25]; however, studies have demonstrated other points of origin, including the gut, lens, and retina [29, 30]. Melatonin is a strong antioxidant that scavenges ROS, decreases MDA, and stimulates the synthesis of antioxidant enzymes [31]. Furthermore, melatonin can protect cultured retinal pigment cells (ARPE-19) from oxidative damage and cell death induced by ischemia and H_2_O_2_ [32, 33]. The protective effect of melatonin on cells against oxidative stress may result from its scavenging of free radicals and the stimulation of the activity of antioxidant defense mechanisms [34, 35]. It is also possible to activate the melatonin membrane receptors MT1 and MT2, which can stimulate the production of a variety of antioxidative enzymes through several signaling pathways [34, 36]. However, the molecular mechanism underlying the effect of melatonin on H_2_O_2_-induced oxidative damage and whether melatonin could induce the autophagic pathway to confer protective effects on RPE cells against damage remain unclear. The aim of this study was to investigate whether melatonin could protect ARPE-19 cells from H_2_O_2_-induced oxidative damage through antioxidative, antiapoptotic, and autophagic mechanisms and to investigate the molecular mechanism underlying the effects of melatonin on ARPE-19 cells.

## 2. Materials and Methods

### 2.1. Reagents and Antibodies

In this study, 3-(4,5-dimethylthiazol-2-yl)-2,5-diphenyltetrazolium bromide (MTT), melatonin, luzindole, paraformaldehyde, bovine serum albumin (BSA), H_2_O_2_, and sodium bicarbonate were obtained from Sigma-Aldrich (Sigma-Aldrich, St. Louis, MO, USA). The cell culture reagents were purchased from Gibco (Grand Island, NY, USA). Primary antibodies against apoptosis-related proteins (Bax, Bcl-2, Cyt c, cleavage-caspase 7, and caspase 7) and autophagy-related proteins (LC3, Beclin-1, mTOR, p-mTOR, ULK1, and p-ULK1) were obtained from Cell Signaling Technology (Danvers, MA, USA). Anti-p62 was obtained from Abcam (Cambridge, UK). Anti-GAPDH, *β*-actin, and rabbit/mouse IgG-horseradish peroxidase antibodies were purchased from Santa Cruz Biotechnology (Santa Cruz, CA).

### 2.2. Cell Culture

The ARPE-19 human RPE cell line was purchased from the American Type Culture Collection (USA). The cells were cultured in Dulbecco's modified Eagle's medium/Ham's F-12 (Corning, Tewksbury, USA) supplemented with 10% fetal bovine serum, 100 *μ*g/mL streptomycin, and 100 U/mL penicillin at 37°C in an atmosphere containing 5% CO_2_. The cells were passaged every 3 days once they grew to approximately 90% confluence.

### 2.3. Cell Viability Assay

Cell survival was tested using the MTT assay. ARPE-19 cells were seeded in 96-well plates at a density of 5 × 10^3^ cells/well for a 24 h incubation process. The cells were treated with vehicle (ethanol) or melatonin at indicated concentrations for 48 h, and they were then treated with 300 *μ*M H_2_O_2_ for 24 h [34, 37] (Figure S1). For the luzindole (melatonin receptor antagonist) test, the RPE cells were seeded as previously detailed. Luzindole was added to the culture medium at a final concentration of 50 *μ*M [34]. One hour later, melatonin was added to the culture medium followed by culturing for 48 h, and H_2_O_2_ was then added followed by culturing for 24 h. Cell viability was evaluated using the MTT assay. Briefly, the culture medium was then removed, and 100 *μ*L of phosphate-buffered saline (PBS) containing 0.5 mg/mL MTT was added, followed by incubation at 37°C for 3 h in the dark. Next, the crystals were dissolved with 100 *μ*L of dimethyl sulfoxide. The absorbance of each well was measured using an Epoch Microplate Spectrophotometer (BioTek, VT, USA) at a test wavelength of 570 nm with a reference wavelength of 630 nm. The relative cell viability is presented as a percentage of cells treated with melatonin compared with those treated with the vehicle.

### 2.4. Lactate Dehydrogenase Release Assay

Cytotoxicity was measured through the lactate dehydrogenase (LDH) [38] release assay conducted using a commercial LDH assay kit (Cayman Chemical, Michigan, USA). In this assay, LDH reduces nicotinamide adenine dinucleotide (NAD) to NADH, which then interacts with a specific probe to produce a color (optical density max = 450 nm).

### 2.5. Thiobarbituric Acid-Reactive Substance Assay

To measure intracellular MDA levels, the ARPE-19 cells were cultured in a 10 cm dish (5 × 10^5^ cells) for a 24 h incubation process. The cells were then added to different concentrations of melatonin for 48 h, after which they were exposed to H_2_O_2_ (300 *μ*M) for 24 h. Thiobarbituric acid-reactive substances (TBARS) were quantified through comparing the absorption result with the standard curve of MDA equivalents generated through acid-catalyzed hydrolysis of 1,1,3,3-tetramethoxypropane.

### 2.6. Apoptosis Assay

Cell apoptosis was measured using an FITC Annexin V Apoptosis Detection Kit (BD Biosciences, Franklin Lakes, USA). Briefly, after treatment, the cells were washed with PBS and incubated in 400 *μ*L of binding buffer containing 2 *μ*L of annexin V-FITC and 2 *μ*L of propidium iodide in the dark for 15 min at room temperature. The stained samples were then analyzed on a FACSCalibur flow cytometry, and the results were analyzed using CellQuest.

### 2.7. ROS Assay

ROS production was determined using a 2′,7′-dichlorodihydrofluorescin diacetate (DCFH_2_–DA) staining assay (Abcam, Cambridge, UK). After melatonin treatment for 48 h, the ARPE-19 cells were incubated with 20 *μ*M DCFH_2_–DA at 37°C for 30 min and then added to 300 *μ*M H_2_O_2_ for 4 h. The ARPE-19 cells were then resuspended in PBS and analyzed through flow cytometry. The percentage of fluorescence-positive cells was recorded on a FACSCalibur flow cytometer using excitation and emission filters of 485 and 530 nm, respectively.

### 2.8. Western Blot Analysis

To determine their protein concentration, the ARPE-19 cells were lysed in immunoprecipitation assay buffer containing a phosphatase inhibitor cocktail tablet and a protease inhibitor mixture (Roche Diagnostics, Basel, Switzerland). The protein concentration was assayed using a bicinchoninic acid assay kit (T-Pro Biotechnology, New Taipei County, Taiwan). Quantified protein lysates (30 *μ*g) were analyzed through sodium dodecyl sulfate-polyacrylamide gel electrophoresis, resolved on 7%–15% polyacrylamide gels. The proteins were then transferred into polyvinylidene difluoride membranes. Each membrane was blocked in 5% BSA for 1.5 h and individually incubated with different primary antibodies against caspase 7, cleavage-caspase 7, Bax, Bcl-2, Cyt c, mTOR, p-mTOR (Ser2448), p-ULK1 (Ser757), ULK1, p62, LC3, Beclin-1, GAPDH, and *β*-actin at 4°C overnight. After being washed three times with Tris-buffered saline mixed with 0.05% Tween-20 (TBST), the membrane was incubated with secondary antimouse or antirabbit antibodies (1 : 10,000; Cell Signaling Technology, MA, USA) for 1 h at room temperature. The signals were detected using a luminescent image analyzer: the Amersham Imager 600 (GE Healthcare Life Sciences, MA, USA). Signal intensities were then quantified using ImageJ.

### 2.9. Immunocytochemistry

ARPE-19 cells were washed three times with PBS and fixed with 4% paraformaldehyde for 30 min at room temperature. The ARPE-19 cells were then washed with PBS and permeabilized with PBS containing 0.025% Triton X-100 for 15 min. The cells were blocked with blocking solution (5% (*w*/*v*) BSA in 1X TBST). After blocking, the antibodies—including anti-LC3 and p62 at a 1 : 250 dilution—were applied, and the cells were incubated at 4°C overnight. Alexa Fluor 594-conjugated rabbit antimouse secondary antibody was prepared in blocking buffer, followed by incubation with the ARPE-19 cells at room temperature for 1 h. After washing, the cells were mounted with 4′,6-diamidino-2-phenylindole-containing mounting medium and analyzed using a fluorescence microscope.

MitoView 633 is a far-red fluorescent dye that stains the mitochondria. The dye is membrane permeable and becomes brightly fluorescent on accumulation in the mitochondrial membrane. After treatment, the medium was removed and added to a prewarmed medium containing diluted MitoView 633. The cells were pelleted and resuspended in medium containing diluted MitoView 633. We conducted the tests at a staining concentration of 200 nM. The cells were visualized using a Zeiss LSM700 confocal microscope.

### 2.10. Statistical Analysis

Each experiment was repeated at least three times, the mean value of the repetitions was calculated, and this value was used in the statistical analysis. The data are presented as the means ± standard deviations (SD). Statistical analyses were performed using one-way analysis of variance (ANOVA) analysis, followed by Tukey's post hoc test which was used to determine the differences between two groups or in multiple groups (GraphPad Prism 6.0; Systat Software, CA, USA). Statistical significance was selected to be *p* < 0.05.

## 3. Results

### 3.1. Melatonin Protects ARPE-19 Cells from H_2_O_2_-Induced Oxidative Damage

**Figure 1:**
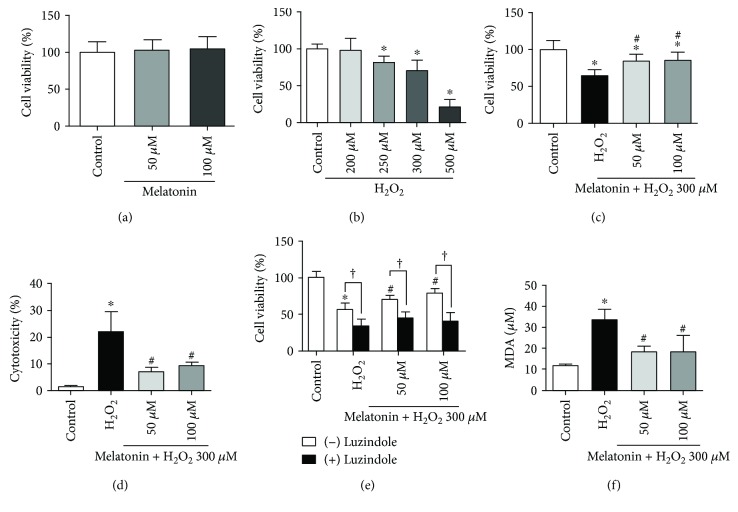
Melatonin protects RPE cells from H_2_O_2_ damage. ARPE-19 cells were treated with different concentrations of melatonin, luzindole (melatonin receptor antagonist), and/or H_2_O_2_. MTT assay was preformed to measure the viability of ARPE-19 cells after melatonin exposed for 48 h (a). MTT assay was preformed to measure the viability of ARPE-19 cells after H_2_O_2_ exposed for 24 h (b). MTT assay was preformed to measure the viability of ARPE-19 cells which pretreatment melatonin for 48 h and then H_2_O_2_ exposed for 24 h (c). Cell cytotoxicity was determined using the LDH release assay (d). MTT assay was preformed to measure the viability of ARPE-19 cells after luzindole exposed for 1 h (e). Lipid peroxidation was measured using the TBARS assay (f). Values are the mean ± SD. ^∗^ *p* < 0.05 versus the control group, ^†^ *p* < 0.05 comparison between cells treated with and without luzindole, and ^#^ *p* < 0.05 versus H_2_O_2_-treated cells.

To examine the cytotoxic effect of melatonin and H_2_O_2_ in cultured RPE cells, the cells were exposed either to 50 or 100 *μ*M melatonin for 48 h or to various concentrations of H_2_O_2_ (200, 250, 300, or 500 *μ*M) for 24 h. The results are presented in Figures 1(a) and 1(b). The viability of the RPE cells was assessed using the MTT assay. As illustrated in Figure 1(a), melatonin at the tested concentrations (50 and 100 *μ*M) was generally safe for the ARPE-19 cells (Figure 1(a)). Treatment of the ARPE-19 cells with H_2_O_2_-reduced RPE cell viability is a dose-dependent manner. Exposure to 300 *μ*M H_2_O_2_ induced approximately 50% cell viability loss (Figure 1(b)). Thus, this concentration (50 and 100 *μ*M melatonin; 300 *μ*M H_2_O_2_) was selected for the subsequent experiments. The ARPE-19 cells were further subjected to melatonin (50 and 100 *μ*M) for 24 h and were then exposed to 300 *μ*M H_2_O_2_ for an additional 24 h to investigate the protective effects of melatonin on the ARPE-19 cells. Statistically, melatonin significantly attenuated the reduction in ARPE-19 viability caused by H_2_O_2_ (Figure 1(c)). Moreover, in order to evaluate the protective effect of melatonin on H_2_O_2_-induced oxidative damage, the study measured LDH release in the ARPE-19 cells following melatonin pretreatment and H_2_O_2_ exposure, and the results showed that H_2_O_2_ treatment induced LDH release, an indicator of cytotoxicity, which was dramatically inhibited by melatonin pretreatment (Figure 1(d)). These results demonstrate that melatonin protects ARPE-19 cells from H_2_O_2_-induced oxidative damage.

### 3.2. Luzindole Decreases Protective Effects of Melatonin on RPE Cells against H_2_O_2_ Damage

We evaluate whether the protective effect of melatonin on H_2_O_2_-induced oxidative damage through the melatonin membrane receptor. Next, the cell cultures were challenged with or without luzindole, a melatonin membrane-receptor antagonist, to determine the direct antioxidant versus receptor-mediated effects of melatonin. Luzindole was added to the culture medium at a final concentration of 50 *μ*M. One hour later, melatonin was added to the culture medium followed by culturing for 48 h, and H_2_O_2_ was then added followed by culturing for 24 h. The ARPE-19 cells cultured with 300 *μ*M H_2_O_2_, to which 50 *μ*M luzindole was added before melatonin, exhibited a statistically significant decrease in viability compared with cells treated with melatonin alone; luzindole blocked the protective effects of melatonin (Figure 1(e)).

### 3.3. Melatonin Inhibits H_2_O_2_-Induced Oxidative Stress in ARPE-19 Cells

**Figure 2:**
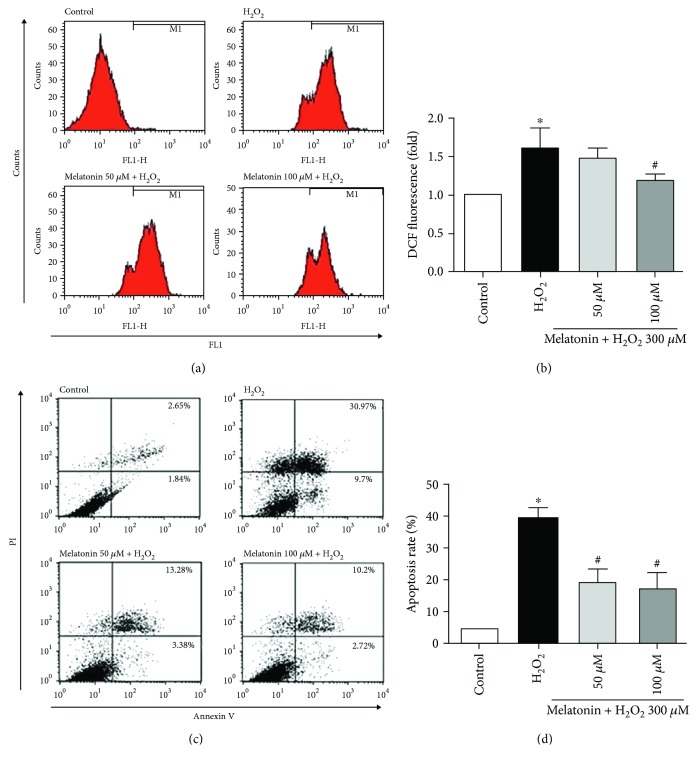
Melatonin inhibits H_2_O_2_-induced ROS production and apoptosis in RPE cells. RPE cells were pretreated with different concentrations of melatonin (50 and 100 *μ*M) for 48 h and exposed to 300 *μ*M H_2_O_2_ for 4 h/24 h. ROS production was determined using the DCFDA assay (a, b). Cell apoptosis was analyzed with PI and annexin V (c, d). Values are the mean ± SD. ^∗^ *p* < 0.05 versus the control group and ^#^ *p* < 0.05 versus H_2_O_2_-treated cells.

To evaluate the effect of melatonin on MDA formation, we examined the level of lipid peroxidation by using the TBARS assay. Treatment with H_2_O_2_ resulted in a significant increase in MDA levels, which was decreased with melatonin pretreatment (Figure 1(f)). As illustrated in Figures 2(a) and 2(b), H_2_O_2_ increased the ROS level in the ARPE-19 cells and melatonin exhibited a statistically significant inhibitory effect on H_2_O_2_-induced ROS production. Thus, melatonin is shown to suppress lipid peroxidation and ROS generation, and this might account for its protective effect.

### 3.4. Melatonin Protects ARPE-19 Cells from H_2_O_2_-Induced Apoptosis

**Figure 3:**
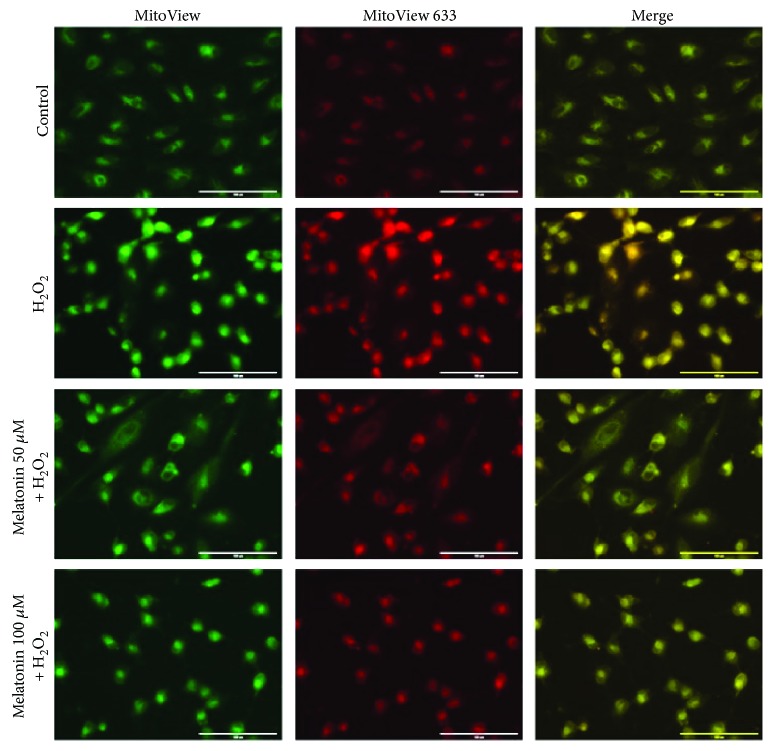
Melatonin inhibits H_2_O_2_-induced apoptosis in RPE cells. RPE cells were pretreated with different concentrations of melatonin (50 and 100 *μ*M) for 48 h and exposed to 300 *μ*M H_2_O_2_ for 24 h. Cell apoptosis was analyzed with MitoView dye. Apoptotic cells were stained red in mitochondria because of the mitochondrial membrane potential changing (scale bars are 200 *μ*m).

To further investigate whether melatonin protects against H_2_O_2_-induced cell death through an antiapoptotic effect, ARPE-19 cell apoptosis was detected using annexin V/PI. As shown in Figures 2(c) and 2(d), the proportion of PI-positive (dead) cells exhibited a statistically significant increase in the ARPE-19 cultures treated with 300 *μ*M H_2_O_2_ for 24 h alone compared with the untreated control cultures. The pretreatment of ARPE-19 cell cultures with 50 and 100 *μ*M melatonin for 48 h before H_2_O_2_ exposure engendered a statistically significant reduction of the proportion of PI-positive (dead) cells compared with cultures exposed to H_2_O_2_ alone. Pretreatment with melatonin reduced H_2_O_2_-induced apoptosis; both concentrations of melatonin had a statistically significant effect (Figure 2(d)). Thus, pretreatment with melatonin can protect ARPE-19 cells from H_2_O_2_-induced cell death. The inhibitory effect of melatonin on ARPE-19 cell apoptosis was further confirmed through testing with MitoView 633. As depicted in Figure 3, the mitochondrial membrane potential increased in the ARPE-19 cells treated with 300 *μ*M H_2_O_2_ for 24 h alone compared with the untreated control cultures. This result suggests that the intrinsic pathway is involved in H_2_O_2_-induced apoptotic cell death. However, the ARPE-19 cells pretreated with 50 and 100 *μ*M melatonin for 48 h before H_2_O_2_ exposure for 24 h exhibited an increased mitochondrial membrane potential compared with the H_2_O_2_ group. This result suggests that melatonin could inhibit H_2_O_2_-induced cell apoptosis in ARPE-19 cells.

**Figure 4:**
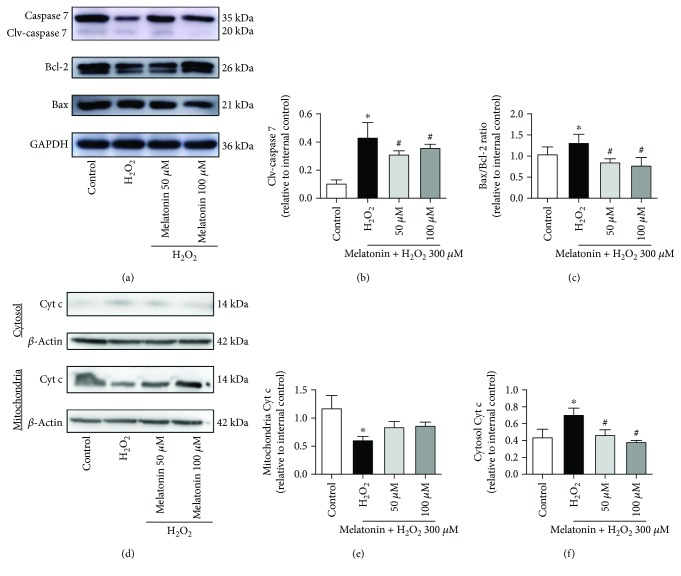
Melatonin inhibits H_2_O_2_-induced apoptosis-related protein expression in RPE cells. ARPE-19 cells were pretreated with the different concentrations of melatonin (50 and 100 *μ*M) for 48 h and exposed to 300 *μ*M H_2_O_2_ for 24 h. Expression levels of apoptosis-related proteins (caspase 7, cleavage-caspase 7, Bcl-2, Bax, and Cyt c) are displayed in (a–f). Protein expression was calculated from densitometry absorbance values of three separate experiments after they were corrected for GAPDH/*β*-actin expression to obtain equal loading. Values are the mean ± SD. ^∗^ *p* < 0.05 versus the control group and ^#^ *p* < 0.05 versus H_2_O_2_-treated cells.

The expression of apoptotic protein results demonstrated that 50 and 100 *μ*M melatonin inhibited H_2_O_2_-induced caspase 7 cleavage in the ARPE-19 cells (Figures 4(a) and 4(b)). We also measured the protein expression of Bcl-2 and Bax to determine whether H_2_O_2_ and melatonin administration engendered any changes. The Bax/Bcl-2 ratio exhibited a statistically significant increase after H_2_O_2_ treatment compared with the control (Figures 4(a) and 4(c)), whereas melatonin achieved a statistically significant reduction of the H_2_O_2_-induced increase in the Bax/Bcl-2 ratio. The Bax/Bcl-2 ratio is critical for regulating the release of cytochrome c from the mitochondria. Therefore, we assessed the level of cytochrome c in the mitochondria and cytoplasm of the ARPE-19 cells. H_2_O_2_ induced a statistically significant increase in cytochrome c levels in the cytoplasm but reduced them in the mitochondria (Figures 4(d)–4(f)). By contrast, melatonin reduced cytochrome c levels in the cytoplasm compared with H_2_O_2_ alone. These results indicate that melatonin protects ARPE-19 cells from H_2_O_2_-induced damage through the antiapoptotic signaling pathway.

### 3.5. Melatonin Activates Autophagic Process in ARPE-19 Cells

**Figure 5:**
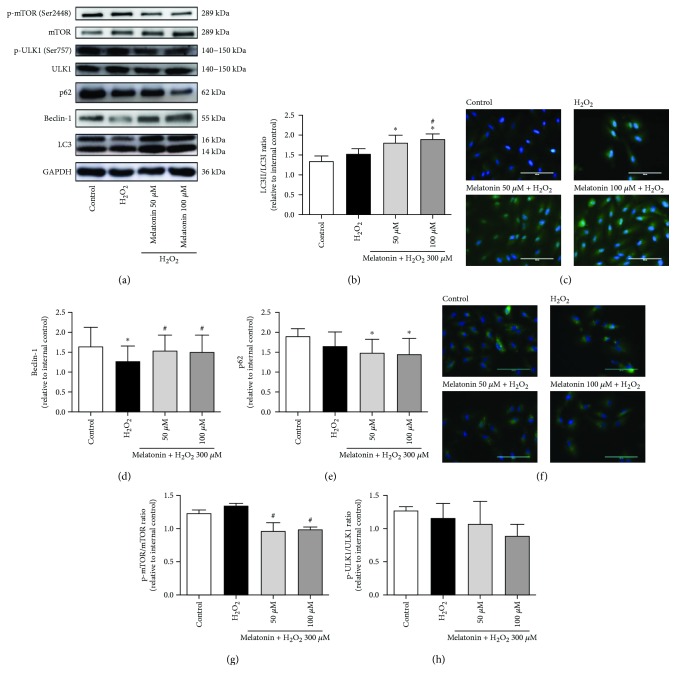
Melatonin increases autophagy in RPE cells. ARPE-19 cells were pretreated with melatonin (50 and 100 *μ*M) for 48 h and exposed to 300 *μ*M H_2_O_2_ for 24 h. Expression levels of proteins are presented in (a–h). Expression levels of LC3 (a, b). LC3 was determined using ICC in ARPE-19 cells (magnification, ×40) (c). Expression levels of Beclin-1 (d). Expression levels of p62 (e). p62 was determined using ICC in ARPE-19 cells (magnification, ×40) (f). Expression levels of mTOR and p-mTOR (Ser2448) (g). Expression levels of ULK1 and p-ULK1 (Ser757) (h). Protein expression was calculated from densitometry absorbance values after correction for GAPDH expression to obtain equal loading. Values are the mean ± SD. ^∗^ *p* < 0.05 versus the control group and ^#^ *p* < 0.05 versus H_2_O_2_-treated cells (scale bars are 100 *μ*m).

A possible protective function of RPE cells against oxidative stress may involve autophagy, which is the major mechanism for renewing all cytoplasmic parts of postmitotic cells; we thus tested for the effect of autophagy. LC3 processing is a classical autophagic marker, and the ratio of conversion from LC3-I to LC3-II is closely correlated with the extent of autophagosome formation. We evaluated the expression of LC3-II autophagic markers. In Figures 5(a)–5(c), it was indicated that pretreatment with 50 and 100 *μ*M melatonin for 48 h before H_2_O_2_ exposure for 24 h in the ARPE-19 cells increased the expression of LC3-II and the LC3-II/LC3-I ratio, thereby providing evidence that melatonin enhances the autophagic process in H_2_O_2_-treated ARPE-19 cells. In the cotreated H_2_O_2_ and melatonin groups, LC3-II levels exhibited a statistically significant increase compared with the H_2_O_2_-treated group (Figure 5(b)). Moreover, Beclin-1 also plays an important role in autophagosome formation and it is therefore considered an autophagic marker for the activation of autophagosomes. We evaluated the expression of the Beclin-1. Beclin-1 levels exhibited a statistically significant decrease in the cells treated with 300 *μ*M H_2_O_2_. Cells cotreated with H_2_O_2_ and melatonin demonstrated a statistically significant increase in Beclin-1 levels compared with H_2_O_2_-treated cells (Figures 5(a) and 5(d)). As displayed in Figure 5(a), melatonin also downregulated the expression of the autophagy-related protein p62 expression. Melatonin caused a statistically significant decrease in p62 levels compared with the control group (Figures 5(e) and 5(f)). As shown in Figure 5(g), the treatment concomitantly reduced mTOR phosphorylation. Cells cotreated with H_2_O_2_ and melatonin demonstrated a statistically significant decreased mTOR phosphorylation levels compared with H_2_O_2_-treated cells. However, melatonin did not cause a statistically significant decrease in ULK-1 phosphorylation levels compared with the H_2_O_2_-treated cells (Figure 5(h)). These findings indicated melatonin pretreatment can increase the autophagic process in ARPE-19 and decrease H_2_O_2_-induced cell death.

## 4. Discussion

Accumulating evidence reveals that the oxidative stress of RPE cells is a crucial aspect of the pathophysiology of AMD [39]. Therefore, studies have focused on designing approaches to protect RPE cells from oxidative stress as therapeutic options for AMD. Exposure to H_2_O_2_ is used as a common model to convey the oxidative stress susceptibility and antioxidant activity of RPE cells [16, 32, 34]. Abundant natural products, particularly flavonoids, confer adaptive survival responses under various adverse environmental conditions through inhibiting oxidative stress [40] [41, 42]. In this study, we observed that the viability of ARPE-19 cells that were exposed to 300 *μ*M H_2_O_2_ decreased by approximately 30%, but melatonin pretreatment significantly inhibited H_2_O_2_-induced damage and increased cell viability. However, MDA reflects oxidative damage in RPE cells. Therefore, to slow the progress and development of early AMD resulting from oxidative damage, it is critical to protect RPE cells by reducing ROS and MDA formation [43–45]. The current study indicated that the exposure of ARPE-19 cells to H_2_O_2_ resulted in increases in ROS and MDA generation, but these effects were significantly ameliorated by treatment with melatonin.

Previous research also reported that secretion levels of melatonin decrease with age, and a particularly demonstrable decrease in circulating melatonin was reported in patients with AMD [28]. Previous studies have demonstrated that melatonin protects RPE cells against blue light and H_2_O_2_ damage *in vitro* and against oxidative stress [32, 34, 35]. Melatonin is a well-known antioxidant and endogenous ROS scavenger and has a higher antioxidant capacity than that of other antioxidants such as vitamin E [30, 46] It may also have protective effects on different types of retinal cells including RPE cells and photoreceptors [46]. In a diabetes study, researchers determined that endogenous and exogenous melatonin may influence metabolic disturbances not only by regulating insulin secretion but also by providing protection against ROS [47]. In the present work, melatonin provides protection against H_2_O_2_-induced ROS. H_2_O_2_-induced RPE cell apoptosis was a well-known study model for drug discovery [16, 48–50]. In the present work, our results demonstrate that H_2_O_2_ can lead to cell apoptosis through increasing apoptosis-related proteins (Bax, Cyt c, and caspase 7) in ARPE-19 cells, but this process was significantly reduced by melatonin. These results suggest that melatonin prevents H_2_O_2_-stimulated cell apoptosis in ARPE-19 cells, which may be strongly related to the antiapoptotic and antioxidative effects of melatonin.

One novel and major finding of this study is that melatonin could reduce H_2_O_2_-induced RPE damage through autophagy. The increased autophagy significantly reduced apoptotic cytotoxicity, suggesting that ROS-activated autophagy was cytoprotective against apoptosis [51]. Autophagy is crucial for the maintenance of homeostasis of RPE cells because it removes dysfunctional organelles and proteins [52–54]. Insufficient digestion because of impaired autophagy in the retinal pigment epithelium causes an accumulation of damaged organelles and toxic proteins, which can contribute to RPE dysfunction and has been associated with the pathogenesis of AMD [55, 56]. Autophagy is regulated by multiple signaling pathways, in which AMPK-mTOR one of the pathways plays an important role in the regulatory process. The mTOR signaling is a negative regulator of autophagy [57]. The level of conversion of LC3-I to LC3-II can be used as an indicator for autophagic activity. The p62 protein is selectively incorporated into autophagosomes through direct binding to LC3-II and efficiently degraded in the autolysosome. Accordingly, the total p62 expression level is negatively correlated with autophagy [58]. Beclin-1 acts during the initiation stage of autophagy by forming the isolation membrane, a double-membrane structure that engulfs cytoplasmic material to form the autophagosome [58]. A previous study indicates a potential function of melatonin in autophagy regulation during oxidative stress [59]. In this study, we determined that melatonin inhibited mTOR and increased autophagic markers (LC3-II and Beclin-1) in unison and promoted autophagy. This finding implies that ROS regulated autophagy in the ARPE-19 cells by downregulating mTOR and upregulating LC3-II and Beclin-1, which increased the potency of melatonin for protecting ARPE-19 cells from oxidative stress-induced cell death. Therefore, melatonin uptake may be beneficial for protecting RPE cells from H_2_O_2_-induced oxidative damage associated with retinal diseases.

We discovered that melatonin mediated autophagy and inhibited H_2_O_2_-induced ROS accumulation and lipid peroxidation in ARPE-19 cells, which could explain the significant protective effect of melatonin on RPE cells. In addition, we discovered these effects of melatonin were blocked by luzindole. Thus, cytoprotection of melatonin might be associated with melatonin receptor-mediated effects, which is consistent with the findings of previous research [34].

## 5. Conclusion

**Figure 6:**
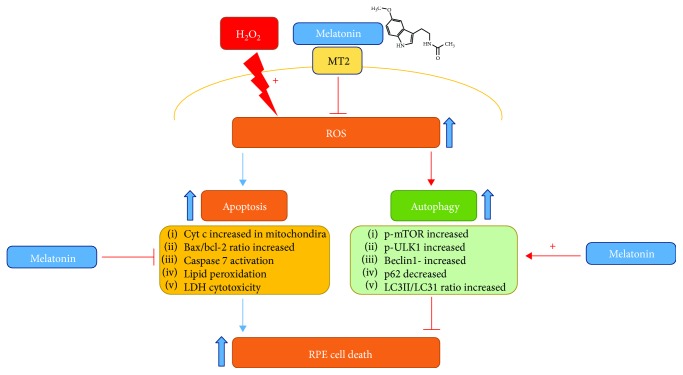
Summary of effects of melatonin against H_2_O_2_-induced oxidative damage in RPE cells. Melatonin inhibits H_2_O_2_-induced RPE cell damage, decreases the apoptosis rate, increases mitochondrial membrane potential, decreases caspase activation, and mediates the autophagy pathway in ARPE-19 cells.

In conclusion, melatonin has protective effects against H_2_O_2_-induced retinal cell death. Melatonin inhibits H_2_O_2_-induced RPE cell damage, decreases the apoptosis rate, increases mitochondrial membrane potential, decreases caspase activation, and mediates the autophagy pathway in ARPE-19 cells (Figure 6). These findings have therapeutic implications for AMD and related inflammatory diseases.

## Data Availability

The data used to support the findings of this study are available from the corresponding author upon request.

## Supplementary Materials

Supplementary Materials Supplementary material contains the experimental protocol in revised articles. Figure S1: the experimental protocol for ARPE-19 cells.
