# Supplementary material for: Protective Effect of Melatonin against Oxidative Stress-Induced Apoptosis and Enhanced Autophagy in Human Retinal Pigment Epithelium Cells
Source: Oxid Med Cell Longev. 2018 Aug 5;2018:9015765. doi: 10.1155/2018/9015765 (PMC6098907; doi:10.1155/2018/9015765)
Supplement: Supplementary Materials — Supplementary material contains the experimental protocol in revised articles. Figure S1: the experimental protocol for ARPE-19 cells. [file 9015765.f1.docx]

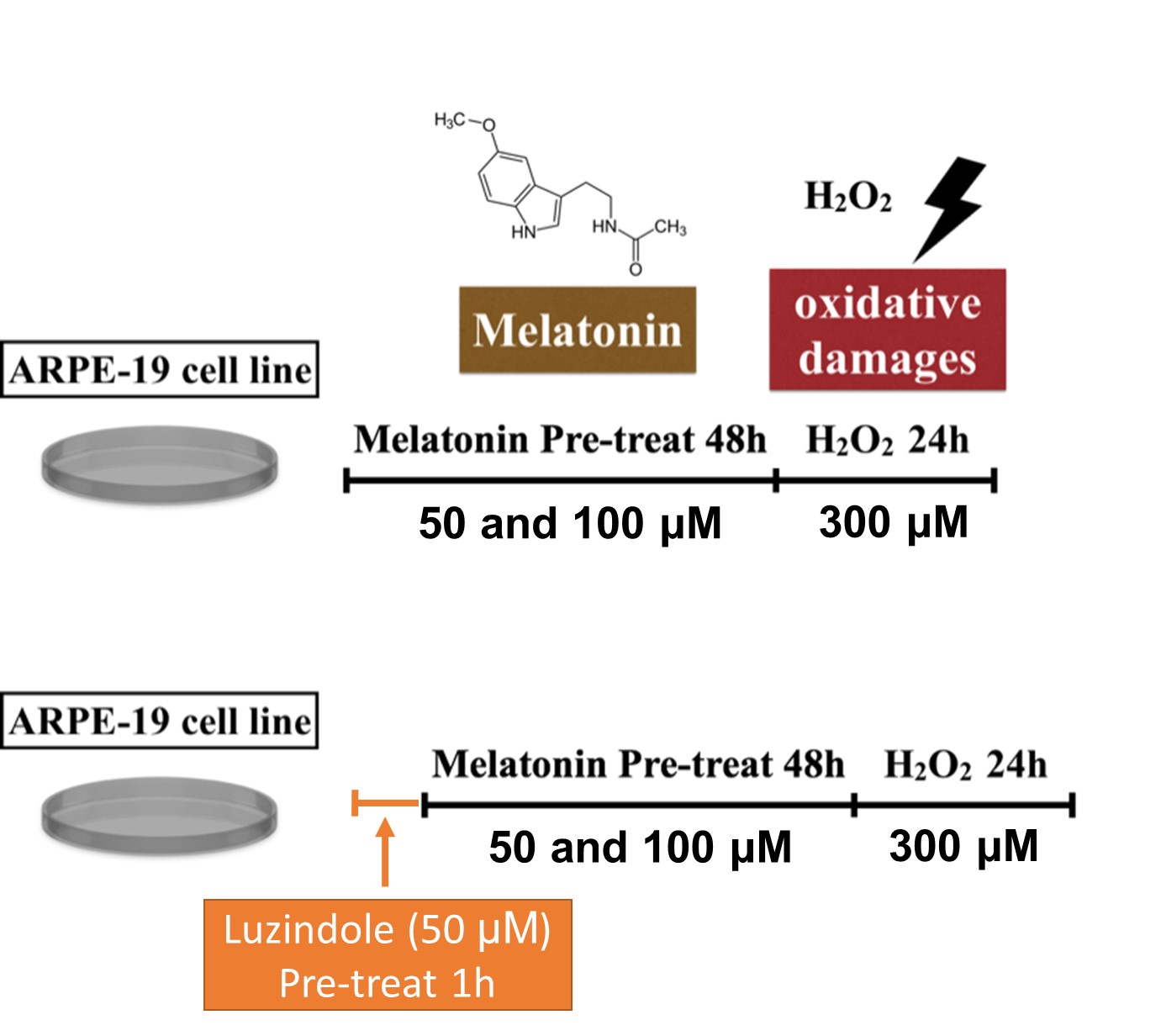


**Figure S1. The experimental protocol for ARPE-19 cell.** The cells were treated with vehicle (ethanol) or melatonin at indicated concentrations for 48 h, and they were then treated with 300 μM H_2_O_2_ for 24 h. For the luzindole (melatonin receptor antagonist) test, the RPE cells were seeded as previously detailed. Luzindole was added to the culture medium at a final concentration of 50 μM. One hour later, melatonin was added to the culture medium followed by culturing for 48 h, and H_2_O_2_ was then added followed by culturing for 24 h.
